# Molecular Characterization of Small Ruminant Lentiviruses of Subtype A5 Detected in Naturally Infected but Clinically Healthy Goats of Carpathian Breed

**DOI:** 10.3390/pathogens9120992

**Published:** 2020-11-26

**Authors:** Monika Olech, Jacek Kuźmak

**Affiliations:** Department of Biochemistry, National Veterinary Research Institute, 24–100 Pulawy, Poland; jkuzmak@piwet.pulawy.pl

**Keywords:** maedi-visna virus (MVV), caprine arthritis encephalitis virus (CAEV), small ruminant lentivirus (SRLV), phylogeny, subtype A5

## Abstract

Small ruminant lentiviruses (SRLVs) are widespread in sheep and goats in Poland, and several subtypes were identified and molecularly characterized up to date. This is the first study that characterizes the molecular properties of A5 strains of SRLV detected in naturally infected, but clinically healthy, Carpathian goats. Segments from three genomic regions (gag, env, and LTR) were analyzed. Genetic distance, pairwise comparison, and phylogenetic analysis revealed that Polish SRLV A5 sequences are closely related to the Swiss and German A5 sequences suggesting a common origin. The epidemiological linkage was identified particularly between the small ruminants of Germany and Poland. Amino acid sequences of immunodominant regions in CA protein were well-conserved within analyzed strains; however, they showed some remarkable changes like substitution (D) to (E), at position 90 in Major Homology Region (MHR) and (T) to (S), at position 141 in epitope 3. In contrast, aa sequences of surface glycoprotein exhibited the highest variability confirming type-specific variation in SU5 epitope. Two deletions in the U3 region of A5 strains were noted: One (8 nt) located near the 5′ end of the U3 region and the other (29 nt) located in the central region of U3. Additionally, all A5 strains had specific deletion (10 nt) in the R region. Furthermore, we did not find a correlation between copies of the CAAAT motif and clinical manifestation in infected animals. These data showed some remarkable features in the viral genome of A5 strains, which may be related to the attenuated phenotype in vivo, characterized by the lack of any clinical signs in infected goats. Certainly, more studies are required to support the hypothesis that these A5 viruses are of low pathogenicity for goats. We want to focus our future studies on the analysis of the whole genomes of these isolates and their biological properties, as well as on clinicopathological studies of goats infected by A5 SRLV, aiming to clarify the pathogenic potential of these viruses.

## 1. Introduction

Caprine arthritis encephalitis virus (CAEV) and Maedi-visna virus (MVV) are two retroviruses classified in the caprine/ovine lentivirus group of the genus *Lentivirus* in the *Retroviridae* family, which can infect goats and sheep under natural conditions. Originally both viruses were believed to be species-specific pathogens, but many studies reported that each of them, following the crossing species barrier, can infect both goats and sheep [1]. In fact, nowadays, they are considered as a single major group of viruses called small ruminant lentiviruses (SRLVs). SRLVs cause the most prevalent lentiviral infection in the world and lead to economic losses and welfare problems in small ruminant production [2]. The monocyte/macrophage lineage and the dendritic cells are the main target cells of SRLV infection, and the virus replication is restricted until the maturation of monocytes to macrophages [3,4].

Animals are mainly infected either by ingesting infected colostrum/milk or by direct contact with infected animals through respiratory secretions [2]. SRLVs induce a persistent infection, which causes a progressive and debilitating inflammatory disease characterized by arthritis, mastitis, and interstitial pneumonia. Sheep usually develop pneumonia with the sporadic occurrence of neurological symptoms, whereas, in goats, the disease is characterized by arthritis in adult animals and encephalitis in kids [5]. There are many pieces of evidence that the susceptibility to SRLV infection varies among different breeds and can be associated with genetic determinants [6,7]. On the other hand, the virulence of SRLV strains can influence the occurrence and severity of the lesions during the course of infection. Virulence of SRLV strains is highly variable, with few highly virulent strains and the majority of strains representing low or even no apparent virulence [8,9,10,11]. In fact, the burden of viruses differs among infected individuals, but both asymptomatic and symptomatic animals can transmit the virus.

Similar to other complex retroviruses, the genome of SRLV is comprised of the *gag*, *pol*, and *env* genes, encoding viral proteins and auxiliary genes, such as the *tat*, *rev*, and *vif*, associated with regulatory functions in virus replication. The provirus genome is flanked by non-coding long terminal repeats (LTRs) regions with of U3 which contains promoter/enhancer sequence, different transcription factor binding sites AP-1, AP-4, AML(vis), GAS, TA, that play a regulatory role in virus transcription and the R and U5 regions [12,13]. The *gag* gene encodes three structural proteins, the capsid (p25CA), the nucleocapsid (p14NC), and the matrix (p16MA) proteins. The largest is the capsid protein, which contains three linear epitopes that elicit a strong antibody response during infection, and for this reason, it is valuable for serological diagnostic tests. Nucleocapsid protein coats the viral RNA genome, while matrix protein ensures the link between the capsid and the envelope [12]. *Pol* gene encodes the enzymes, protease (PR), dUTPase, integrase (IN), and reverse transcriptase (RT), which are involved in replication and integration of proviral DNA into the host cell DNA. Finally, *env* encodes the surface (gp135SU) and the transmembrane (gp46TM) glycoproteins. SU of SRLV has many important biological functions and contains domains that are responsible for the recognition of host cell receptors. Moreover, SU stimulates the production of antibodies and is genetically variable, determining the antigenic variability of the different subtypes [14]. TM, which allows the fusion of the viral envelope and the membrane of the host, is a much more conserved protein and is a good candidate to be used in ELISAs [15].

SRLVs are divided into five genetic groups, A-E, and further divided into several subtypes. Subtype B1 is considered as the prototypic caprine arthritis encephalitis virus (CAEV), while subtype A1 is the prototypic maedi visna virus (MVV). Genotypes A and B are widespread throughout the world, however, some subtypes appear to have a relatively restricted distribution, which may be related to domestication pathways of small ruminants [16]. Many studies have documented the circulation of different types/subtypes of SRLVs in both sheep and goats due to cross-species infection, which is a typical hallmark of SRLV infection over the world. In fact, the majority of subtypes can cross the species barrier between sheep and goats under field conditions [1,17,18].

SRLVs adapted to a new host may acquire novel biological and pathogenic properties like extended cell and/or tissue tropisms and increased virulence, which may threaten the health of other animal species [19]. On the other hand, viruses that crossed the species barrier may become less pathogenic and virulent or even attenuated, as was demonstrated for SRLV subtype A4 isolated from asymptomatic goats [20]. Therefore, the study of the risk of cross-species infection with SRLV and the characterization of molecular and biological properties of new viruses becomes an emergency.

SRLVs isolated so far from sheep and goats in Poland belonged to the well-known subtypes B1, B2, and A1, as well as to the more recently established subtypes A12, A13, A16, A17, and A18 [21,22,23]. In this report, for the first time, we described SRLV subtype A5 sequences from Polish goats of the Carpathian breed. We determined their partial *gag*, *env*, and LTR sequences, and investigated their relationships with other known SRLV isolates.

## 2. Results

### 2.1. Phylogenetic Analysis of SRLV Based on Gag Fragment

**Figure 1:**
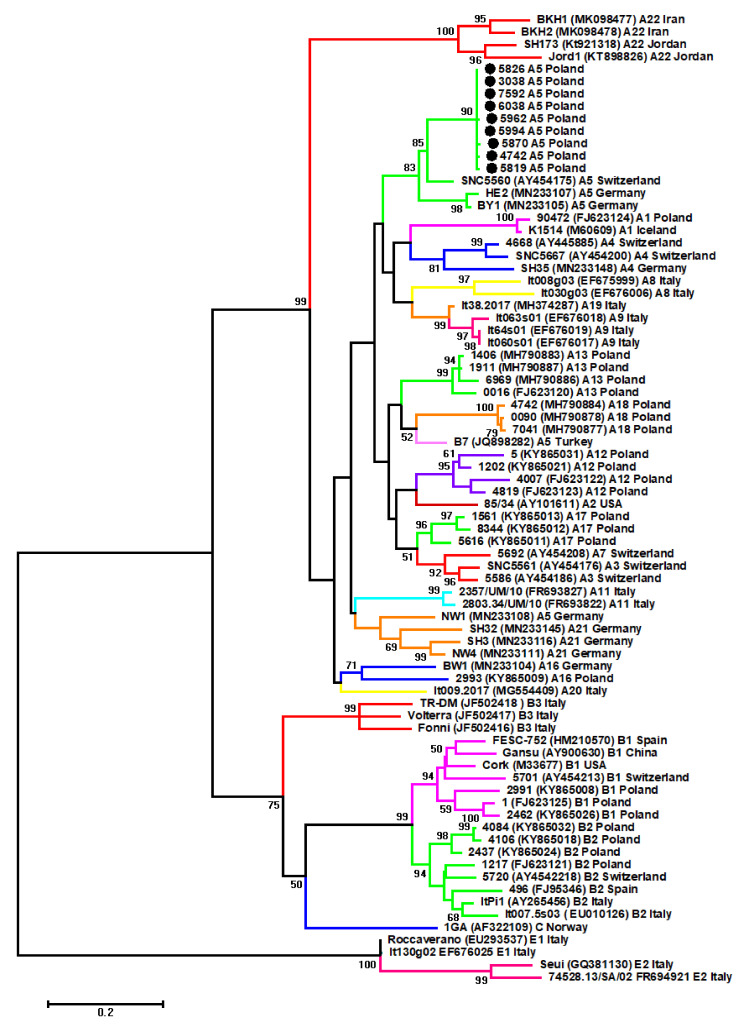
Maximum-likelihood phylogenetic tree based on the alignment of *gag* sequences (423 bp) from 77 sequences: Nine analyzes in this study sequences (labeled by black circles), and 69 reference small ruminant lentiviruses (SRLVs) strains originated from different geographical areas. Strains are shown by name followed by GenBank accession number, genotype, and country of origin. Numbers at the branches indicate the percentage of bootstrap values obtained from 1000 replicates. Bootstrap values > 50% are shown.

All animals were seropositive, and nine samples which gave strong *gag* PCR product were sequenced and then subjected to phylogenetic analysis. To determine evolutionary relationships between the analyzed sequences and other SRLV sequences representing SRLV genotypes described to date, maximum-likelihood (ML) phylogenetic tree based on the alignment of *gag* nucleotide sequences was constructed. The ML tree revealed that all analyzed sequences (#5819, #4742, #5826, #5870, #6038, #5994, #5962, #7592 and #3038) were closely related to each other and all belonged to subtype A5 (Figure 1). 

The affiliation was supported by a high bootstrap value (83.0%). Sequences were clustered together with the sequences of Swiss strain 5560 and German strains, HE2 and BY1. However, sequences of German strain NW1 and Turkish strain B7, which were previously affiliated to subtype A5 [16,24], in our analysis grouped with other subtypes, subtype A21 and A18, respectively. Overall tree topology constructed using Bayesian method was consistent with the topology of ML tree, supporting affiliation of Polish sequences into subtype A5 (data not shown).

### 2.2. Genetic Distance and Pairwise Comparison

The analyzed samples were also used to amplify the V4/V5 fragment of the *env* gene and U3-R fragment of the LTR region. The LTR fragments were successfully amplified from all nine samples, while the *env* fragments were obtained from seven samples. The percentage of nucleotide substitutions were determined by pairwise comparison, using consensus sequences. All nine Polish SRLVs A5 sequences were found to be closely related to each other, showing an average degree of variability of 0.3% for *gag*, 1.6% for LTR, and 2.6% for *env* sequences with a range of variability of 0 to 0.9%, 0 to 2.8% and 1.8 to 3.8%, respectively. The mean genetic distance between Polish SRLV A5 sequences and sequences of other A5 strains (5560, BY1, and HE2) was 10.1 %. Generally, Polish SRLV A5 sequences were closely related to the sequence of Swiss strain 5560 with mean genetic distance of 8.4% and appeared to be more moderately related to the sequence of German strains HE2 and BY1 with mean genetic distance of 11.0% (Table 1). 

The mean genetic distance of Polish SRLV A5 sequences and sequences of Germain strain NW1 and Turkish strain B7 was 14.4% and 13.2%, respectively. The mean genetic distance between Polish A5 sequences and sequences of other subtypes representative of genotype A varied from 14.4% to 18.5% (Table 1). Based on phylogeny, Polish SRLV A5 sequences were also similar to sequences from subtype A19 (14.5%), A17 (14.4%), and A3 (14.7%).

**Figure 2:**
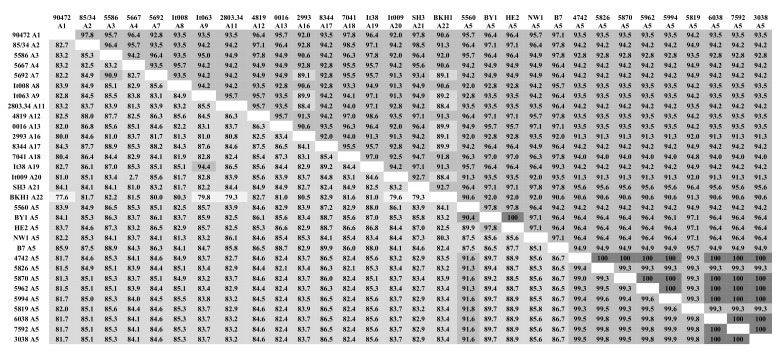
Pairwise percent identity of *gag* nucleotide and amino acid sequences of Polish SRLV A5 sequences compared to different subtypes of genotype A. The lower matrix shows the percent identity of nucleotide sequences, and the upper matrix shows the percent identity of amino acid sequences. The intensity of the color is correlated with pairwise percent identity.

Pairwise percent identity of the *gag* nucleotide and amino acid sequences between Polish SRLV A5 sequences and sequences of strains representing different subtypes were performed. The analysis revealed that Polish A5 sequences shared significant-high sequence identity with each other, ≥99.0% nucleotide sequence identity, and ≥99.3% amino acid sequence identity (Figure 2). The nucleotide sequences of the #7592, #3038, and #6038 were identical. This analysis also showed that despite some nucleotide sequence variabilities almost, all Polish SRLV A5 sequences shared 100% amino acid sequence identity. The only exception was a sequence of #5819 strain, which shared 99.3% amino acid sequence identity, indicating that their nucleotide substitutions were non-synonymous mutations. Consistent with phylogenetic analysis, Polish SRLV A5 nucleotide sequences showed the highest sequence identity with Swiss strain 5560 (91.3–91.6%). With A5 strains, BY1 and HE2, sequence identity was also high and ranged from 89.2% to 89.7% and from 88.5% to 88.9%, respectively. Furthermore, Polish SRLV A5 sequences shared 86.5–86.7% and 85.3–85.8% nucleotide sequence identity with strains B7 and NW1, respectively. Interestingly, Polish SRLV A5 shared 96.1–97.1% amino acid sequence identity with BY1, HE2, B7, and NW1 A5 strains and only 94.2% with strain 5560. Nucleotide and amino acid sequence identity between Polish SRLV A5 strains and strains from other subtypes were 81.5–86.5% and 90.65–94.2%, respectively. The highest nucleotide and amino acid sequence identity was noted with strains representing subtype A2 (84.6–85.1% and 94.2–94.9%), A3 (85.1–85.3% and 92.8–94.9%), A8 (84.9–85.3% and 93.5–94.9%), A17 (86.0–86.5% and 94.2–94.9%) and A19 (85.1–85.6% and 94.2–94.9%).

### 2.3. Comparative Analysis of Immunodominant and Genetic Regions

**Figure 3:**
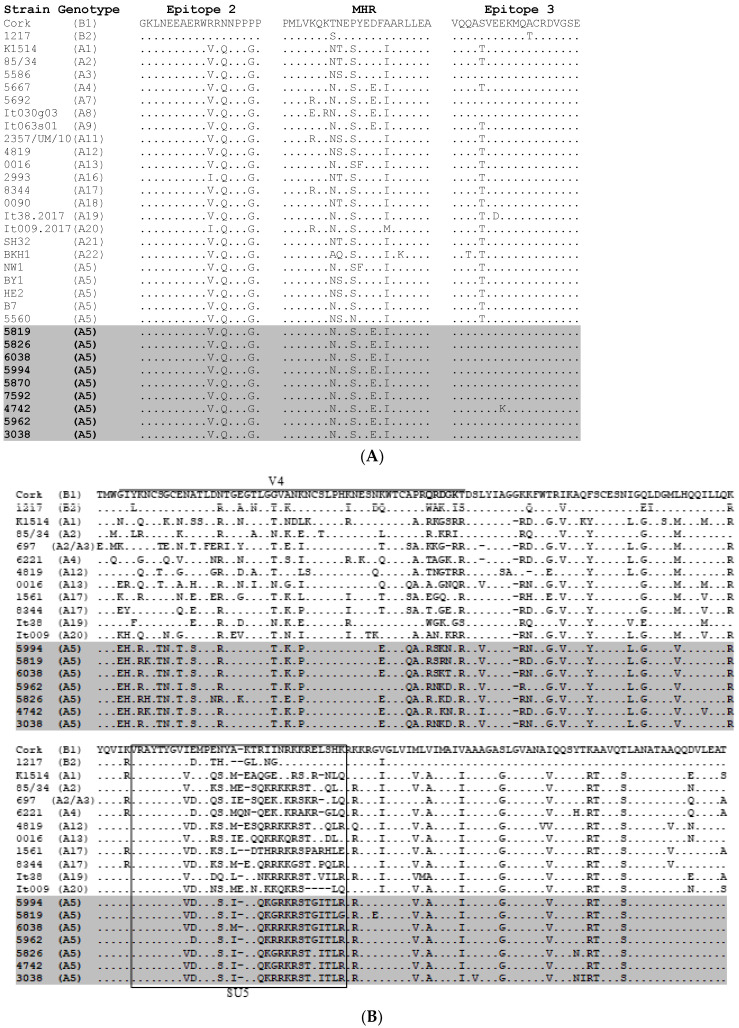
Sequence homology of Gag (**A**) and Env (**B**) immunodominant regions between Polish SRLV. A5 strains were analyzed in this study and the reference strains from different genotypes. Deletions are indicated by a dash (-), and identical residues are indicated by dots (.).

To analyze the sequence conservation of both the Gag and SU regions of Polish A5 strains, the 625 bp fragment of *gag* gene spanning immunodominant regions of capsid protein (CA), and the 608 bp fragment of *env* gene spanning N-terminal part of surface glycoproteins (SU) were amplified, cloned and sequenced. The deduced amino acid sequences were aligned with the corresponding reference sequences of strains belonging to known subtypes of genotype A and genotype B (Figure 3). 

CA sequences of Polish A5 SRLV differed from 0% to 1.4% to each other and showed the presence of asparagine-valine (NV) motif, like it was found in other strains from genotype A, instead of glycine-glycine (GG) motif, which was typical for B-type strains (data not shown). The immunodominant epitope 2 was almost identical in all A5 strains like in other strains representing group A, except strains of subtype A16 and A20, which had a substitution of V (valine) to I (isoleucine). All Polish SRLV A5 sequences had a substitution of aspartic acid (D) to glutamic acid (E) in the major homology region (MHR) and threonine (T) to serine (S) at position 141 within epitope 3. The same substitution in MHR and immunodominant epitope 3 was also observed in strains belonging to subtypes A4, A7, A8, A9, and in A7, A8, respectively. The substitution of lysine (K) to glutamic acid (E) at position 144 in epitope 3 was exclusively found in Polish A5 for strain #4742.

The SU sequences of Polish SRLV A5 strains were more heterogeneous than CA sequences and differed from 2.1% to 6.2%. Variable region (V4) was well-conserved in all A5 sequences, however, it differed prominently from other strains representing known subtypes. Most of the amino acid substitutions were present in the hypervariable region (HV2) (QRDGK, according to the Cork sequence) [25]. Comparison of aa sequences in SU5 epitope revealed a perfectly conserved motif located at the N-terminal part (VRAYTYGV) and a highly variable motif at the C- part of the epitope. This motif (ESYI/MKTQKG/RRKRSTG/EITL) was well-conserved among strains belonging to subtype A5, however, it differs significantly from aa sequences of other subtypes (Figure 3B).

To determine the rate of evolution in nucleotide sequences encoding Gag and Env proteins, two values, dN (rate of non-synonymous substitutions per non-synonymous site) and dS (rate of synonymous substitutions per synonymous site), were estimated. The results showed that dN/dS ratio was 0.208 and 0.436 for *gag* and *env* sequences, respectively, showing purifying selection.

**Figure 4:**
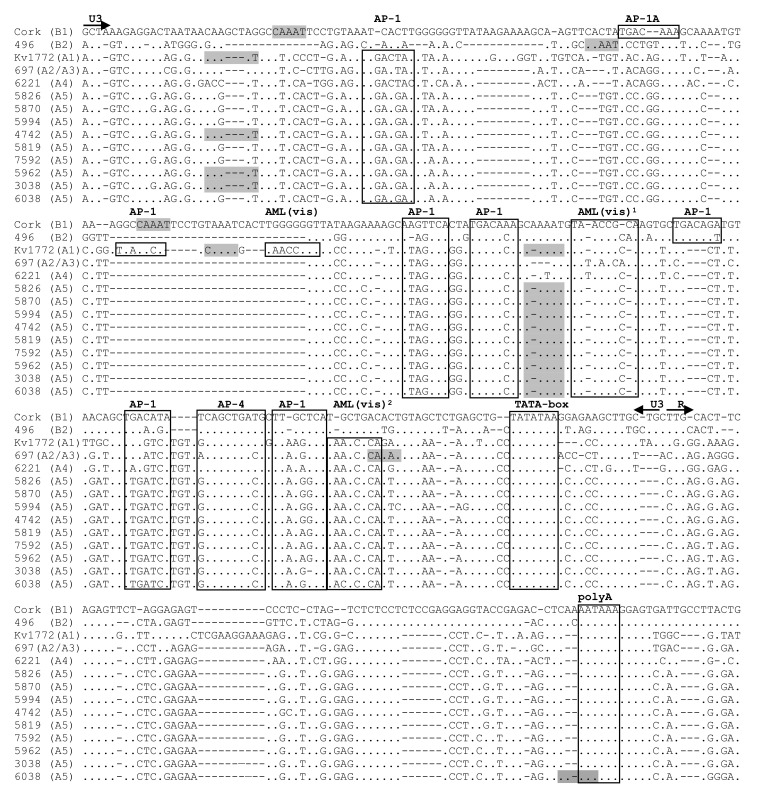
Alignment of U3-R sequences of the long terminal repeats (LTR) region from Polish A5 SRLV strains. Sequences were aligned against virulent B1 strain Cork, neurovirulent A2/A3 strain 697, neurovirulent A1 strain Kv1772, arthritic B2 strain 496, and attenuated A4 strain 6221. Dots indicate identity with Cork, and dashes represent gaps. The boundaries between U3, R, and U5 are indicated by straight arrows. AP-1, AP-4, AML (vis) motifs, the TATA box, and polyadenylation signal (poly A) are marked by boxes. The grey boxes represent CAAAT sequences.

LTR nucleotide sequences of Polish SRLV A5 strains were aligned with the corresponding sequences of SRLV strains of defined virulence. A5 sequences were compared to virulent B1 strain Cork [26], A2/A3 strain 697 isolated from sheep with neurological symptoms [11], neurovirulent A1 strain Kv1772 [8], B2 strain 496 isolated from arthritic sheep [19], as well as to the attenuated A4 strain 6221 [27]. The alignment of LTR nucleotide sequences showed high genomic heterogeneity (Figure 4). 

The LTR sequences of all A5 strains were highly homogenous to each other, but quite distant from the other sequences. However, the sequences corresponding to the TATA-box, AP-4, and the polyadenylation signal (poly A) were quite conserved among all strains. A5 LTR presented two highly conserved AML(vis) sites. Moreover, AML(vis)^2^ sequence close to the TATA box was present only in MVV-like sequences. The AP-1 sites were less conserved. We identified different sequences for AP-1 site which occurred in CAEV-like strains (TGACAAA, TGACAGA, TGACATA, TTGCTCA, AAGTTCA, TGACACA, TGACAGT, TGAAAGA, TTGCTCA) and MVV-like sequences (TGACTAT, TGAGTCA, TTAAGTCA, TGACACA, ATAGTCA, ATAGTCA, TGACAAA, TGAATCA, TTAGGTCA, TGACTAC, TAAGTCA, TTAGATCA). In A5 strains, we observed five different sites of AP-1 (TGAAGAT, ATAGTCA, TGACACA, TTGATCA, TTAGG/AG/TCA). Two deletions in the U3 region of A5 strains were noted; one (8 nt) located near the 5′ end of the U3 region and a second one (29 nt) located in the central region of U3. The presence of these deletions besides the A5 strain has also been shown in attenuated strain 6221, as well as in virulent strains 697 and 496. All A5 strains also had deletion (10 nt) in the R region. The same deletions were present in all reference strains except for strain Kv1772 (A1). The number of repeats of the CAAAT sequence was diverse depending on the strains. Strain Kv1772 had three copies of the CAAAT sequence, while Cork, as well as strains #6038, #3038, #4742, and #5962, had two copies. Strain #5994, #5819, #5826, #5870, #7592, and strain 496 (B2) and 497 (A2/A3) had only one copy of the CAAAT sequence, while strain 6221 representing subtype A4 had no copies.

## 3. Discussion

SRLVs are widespread in sheep and goats in Poland and to date, several subtypes have been identified and molecularly characterized [21,22,23]. It is obvious that, over time, more new SRLV subtypes could have been introduced by importing animals, management practices, or through other routes like cross-species infection, genetic drift, and the formation of quasi-species or genetic recombination. Therefore, it is quite clear that detailed knowledge about the genetic diversity of SRLV and the genotypes circulating in the field is crucial for the effectiveness of control programs, as well as for the development of diagnostic tests for the successful detection of local strains. 

In this study, we characterized the Polish SRLV A5 sequences detected in naturally infected, but clinically healthy Carpathian goats. This is the first time, to our knowledge, that A5 subtype SRLV was found in goats from Poland, even though many sheep and goats from numerous flocks were tested for SRLV so far. The phylogenetic characterization of these viruses, based on *gag* gene sequences, revealed their close clustering together with Swiss (strain 5560) and German (strains BY1 and HE2) A5 strains, suggesting their common origin. Consistent with phylogenetic analysis, sequences of Polish SRLV A5 strains showed the highest identity with strain 5560 (91.3–91.6%), BY1 (89.2–89.7%), and HE2 (88.5% to 88.9%). However, the differences between them and the strains representative of other subtypes of SRLV from group A ranged from 14.4% to 18.5%, which was quite similar to the intersubtype divergences within this group.

Detection of subtype A5 in Germany [16], a country neighboring Poland, suggests evolutionary and epidemiological linkages between small ruminants from both countries. It can be supposed that SRLVs were introduced to Poland through the movement of sheep together with German settlers during the Second World War [16,23,28]. Co-circulation of subtypes A5 and A21 in German flocks [16] and the clustering of Germain A5 NW1 sequences together with A21 sequences (this study), support their proposed relationship. Moreover, subtype A21, the most common subtype in Germany, is genetically similar to subtypes A12 (84.9% nucleotide and 96.4% amino acid sequence identity), A13 (83.2% nucleotide and 94.9% amino acid sequence identity), A17 (84.9% nucleotide and 97.0% amino acid sequence identity) and A18 (84.9% nucleotide and 97.0% amino acid sequence identity), which were exclusively found in sheep and goats from Poland. Therefore, it seems plausible that the animals’ movement during the Second World War might be a suitable factor for the broad transmission of SRLV. This would also imply the existence of a common ancestor for these SRLV subtypes in both countries.

The study of Molaee et al. [16] and Muz et al. [24] suggested that the ancestors of European SRLV, especially for genotype A, are those found in Turkey and that the evolution of some subtypes of SRLV reflex domestication pathways of small ruminants. Accordingly, the subtype A5 was found in Turkey [24] and Germany [16] as start and end terminals, respectively, as well as in Slovenia [29] and Switzerland [30], the countries lying on the route between Turkey and Germany. The Carpathian goat is an ancient breed that evolved in southeastern Europe, including Romania and Poland. However, there is no information about the subtypes of SRLV circulating in Romania, but we cannot exclude that Carpathian goats or local primitive breeds of goats, being the progenitor of Carpathian goat that occupied large areas of the Carpathian Highland, may have become infected in the past.

Analysis of deduced amino acid sequences revealed a high degree of Gag protein conservation in strains from genotype A, especially within immunodominant epitopes 2 and 3. These epitopes are important for maintaining cross-reactivity in serological tests based on Gag-derived antigen [31,32]. Apart from immunodominant epitopes, the major homology region (MHR) is usually well-conserved in many retroviruses, and mutations in this region may impair capsid assembly and reduce infectivity of HIV-1 [33,34]. It is not known whether substitution (D) to (E) in MHR that was specific for Polish A5 subtypes and subtypes A4, A7, A8 can lead to a similar event.

As expected, the SU sequences exhibited higher variability than CA [35,36]. While aa sequences were well-conserved within A5 strains, the divergences between other strains were particularly evident for variable region V4. Notably, it was seen for hypervariable region QRDGK, known as the highly variable region (HV2) [25]. This region undergoes rapid sequence variation, which may be important in virus–host interactions. It has been postulated that V4 is structurally and functionally analogous to the V3 domain of HIV-1 [37], and like V3, it can determine cellular tropism and synthesis of neutralization antibodies [38]. We can only speculate that aa changes in this region may account for different features of A5 strains, like what was seen for V3 of HIV and FIV [39,40]. Our study also indicated, that immunodominant epitope SU5 retained the conserved amino-terminal part (VRAYTYGV), however, it expressed a highly variable motif at the C- part, which was quite well-conserved among A5 strains. It was shown that the SU5 epitope is responsible for type-specific immune response allowing strain-specific diagnosis [38,41]. In this study, we found some sera from goats infected with A5 subtype that failed to react with the SU1/SU5 antigens, derived from other subtypes developed in the previous study [22] (data not shown), most probably because of the significant antigenic divergence within SU5 domain. This can be an additional argument highlighting the usefulness of the test with homologous SU5 protein for tracking SRLV subtypes.

LTR-U3-R region of lentiviruses contains promoter sequences and transcription factor binding sites important for virus transcription and replication. Several previous reports showed that mutations, deletions, or insertions in LTR of different SRLVs had an impact on their virulence [8,9,42,43,44,45,46]. The fact that none of the goats analyzed in this study has shown any clinical symptoms may suggest that A5 viruses may be apathogenic for these goats. Therefore, LTR nucleotide sequences of Polish SRLV A5 strains were aligned with other SRLV sequences of strains with defined virulence. The analysis showed well-conserved sequences of AP-4 site and highly conserved sequence of TATA-box and Poly-A site in all strains, confirming the importance of these sites for virus replication [47]. Identification of different AP-1 sites confirmed previous findings, which may indicate that AP-1 sites may be functional even despite some mutations [19,48,49]. It was shown that the strains with deletions in U3-R region characterized slow virus replication in vitro and lower activity of the promoter [8,9,10]. In this study, the sequences of the U3 region of LTR of A5 strains lacked the sequence repeats that were present in LTR of virulent Kv1772 and Cork strains. However, the presence of these deletions besides A5 strains has also been shown in attenuated strain 6221, as well as in neurovirulent strain 697 and arthritic strain 496. Moreover, a correlation between a deletion in the R region and the occurrence of clinical signs also has been suggested [19,42]. In the present study, the LTR of Polish SRLV A5 strains contained a 10 nt deletion in the R region. However, the same deletion was present in sequences from asymptomatic strain 6221, as well as in viruses isolated from the clinically affected animals. Furthermore, we did not find a correlation between the number of CAAAT motif copies and clinical manifestation in infected animals, which was suggested by some authors [8]. LTR of A5 strains carries one or two copies of the CAAAT motif similar to highly virulent Cork, 496 and 497 strains. Overall, this shows a low association of pathogenic potential and LTR sequence and suggests that other factors can be responsible for the lack of clinical symptoms [19,24,42,45]. It is also possible that mutations and deletions in the LTRs may only co-exist with mutations in other fragments of the SRLV proviral genome, which in fact may lead to the reduction of pathogenicity.

Altogether, this study confirmed for the first time the occurrence of subtype A5 SRLV in goats from Poland and their relationship with known SRLV isolates. Polish SRLV A5 strains were closely related to Swiss and German A5 strains suggesting their common origin. Especially, the epidemiological linkage between the small ruminants of Germany and Poland should be taken into account in such analysis. Polish SRLV A5 strains showed some remarkable features in their genome, which may reflect their evolution, adaptation to a new host, and perhaps, can be related to the attenuated phenotype in vivo. Certainly, more studies are required to support the hypothesis that these A5 viruses are pathogens with low pathogenicity for goats. We want to focus our future studies on the analysis of the whole genomes of these isolates and their biological properties, as well as on clinicopathological studies of goats infected by A5 SRLV aiming to clarify the pathogenic potential of these viruses.

## 4. Materials and Methods

### 4.1. Animals and Samples

The study was performed in one flock, counting 20 goats representing an old local Carpathian breed. The flock was located in the Malopolska region, which belongs to the Carpathian region in the south of Poland. The Carpathian goat is an ancient breed present in Carpathian Mountain in Poland in the 19th and 20th century which later, became an extinct breed. In 2005, however, very small flocks of these goats were spotted in Poland, and were moved to the National Research Institute of Animal Production in Cracow. Over the last 10 years, this flock has been recreated, and currently, it is covered by the genetic resources protection program, supported by the Ministry of Agriculture and Rural Development. In 2018 all adult animals belonging to this flock were serologically tested, and they showed close to 100% seropositivity. However, none of them exhibited any clinical signs of SRLV infection. Very high seroprevalence and the existence of old local breed without crossbreeding, which is quite frequent in other flocks from this region, prompted us to investigate the molecular nature of SRLV infecting goats from this flock. Blood was taken by jugular venipuncture and collected in EDTA and serum tubes for further analysis. Peripheral blood leukocytes (PBLs) were used for DNA isolation, as previously described [22]. At the time when blood was taken, none of the goats exhibited any clinical signs of SRLV infection. All methods were performed in accordance with the relevant guidelines and regulations. Specifically, blood collection was approved (no 37/2016) by the Local Ethical Committee on Animal Testing at the University of Life Sciences in Lublin (Poland).

### 4.2. Serological Study

The serological status of SRLV infection was determined using the ELISA test (ID Screen MVV/CAEV Indirect Screening test, IDVet, Grabels, France).

### 4.3. PCR Amplification and Sanger Sequencing

For initial screening of provirus-positive goats, nested PCR amplification of the 625 bp fragment of the *gag* gene was performed using DNA samples from 20 goats. For molecular characterization of SRLV, we selected DNA samples from nine goats, which gave strong *gag*-specific PCR amplification. Besides amplification of the *gag* gene fragment, the V4/V5 (608 bp) fragment of the *env* gene and the U3-R fragment of the LTR region were amplified by nested PCR as described elsewhere [4,22]. PCR products were analyzed by electrophoresis on 2% agarose gel containing ethidium bromide (1µg/mL) in 1 × TAE buffer. PCR products were purified (NucleoSpin Extract II kit, Macherey Nagel GmbH & Co KG, Düren, Germany) and cloned into the pDRIVE vector (Qiagen GmbH, Hilden, Germany). Ligation products were used to transform EZ Competent Cells (Qiagen), and plasmid DNA was extracted using the NucleoSpin Plasmid kit (Marcherey-Nagel GmbH & Co KG, Düren, Germany). Five clones derived from each DNA sample were sequenced on a 3730xl DNA Analyzer (Applied Biosystems, Foster City, CA, USA) using Big Dye Terminator v3.1 Cycle Sequencing kit. To control for PCR errors, both sense and antisense strands were sequences.

### 4.4. Bioinformatic Analysis

The obtained SRLV sequences were trimmed and analyzed using the Geneious Pro 5.3 software (Biomatters Ltd., Auckland, New Zealand). The consensus sequences corresponding to each amplified fragment were generated from each sample. All novel sequences reported in this study were submitted to the Gen-Bank database under accession numbers: MT360654-MT360678. The evolutionary relationships of analyzed strains with other published sequences were investigated by constructing the phylogenetic trees from multiple alignments of the *gag* region. The available sequences of the reference SRLV strains of genotypes A-C and E, representing isolates from many countries, were included in the analysis. The sequences representing subtypes A6, A10, and A15 had to be excluded from the analysis, since their *gag* sequences are not available. Additionally, strains representing subtype A14 were not included because of the shortness of the *gag* sequences. In the present study, the SRLV found by *Colitti* et al. [50] were renamed from A18 to A19 and from A19 to A20. All sequences were aligned using MUSCLE. Model testing was performed to select the best evolutionary model based on the Bayesian information criterion (BIC) and Akaike information criterion (AIC). According to the results, Hasegava-Kishino-Yano (HKY) model with the gamma distribution ( + G) with five rate categories and by assuming that a certain fraction of sites are evolutionarily invariable ( + I) was applied to infer a phylogenetic tree using maximum likelihood (ML) method. Nonparametric Bootstrap analysis with 1000 iterations was used to evaluate the robustness of evolutionary relationships. Alignment, model testing, and ML tree building were performed using MEGA 6 software [51]. The tree topology was confirmed using the Bayesian method with the HKY model implemented in Geneious Pro 5.3 software (Biomatters Ltd., Auckland, New Zealand). 

To evaluate the nucleotide and amino acid sequence percent identity (percentage of bases/residues which are identical), pairwise comparison of the Gag sequences between the Polish SRLV A5 isolates and selected isolates of other subtypes from other geographic regions was performed using Geneious software.

Pairwise genetic distances were estimated with the MEGA 6 software [51] according to the p-distance substitution model with default settings. Non-synonymous and synonymous substitution rate was determined using SNAP (Synonymous Non-synonymous Analysis Program) v 2.1.1. implemented in the Los Alamos National Laboratory HIV-sequence database (https://www.hiv.lanl.gov/content/sequence/SNAP/SNAP.html).

## Figures and Tables

**Table 1:** Mean nucleotide distance of Polish SRLV A5 *gag* sequences analyzed in this study (shown in bold) compared to other A5 sequences (5560, HE2, BY1, NW1, B7) and other subtypes of genotype A (inter-genotype).

	A1	A2	A3	A4	A7	A8	A9	A11	A12	A13	A16	A17	A18	A19	A20	A21	A22	5560	HE2	BY1	NW1	B7
A1	-	-	-	-	-	-	-	-	-	-	-	-	-	-	-	-	-	-	-	-	-	-
A2	17.2	-	-	-	-	-	-	-	-	-	-	-	-	-	-	-	-	-	-	-	-	-
A3	16.1	13.9	-	-	-	-	-	-	-	-	-	-	-	-	-	-	-	-	-	-	-	-
A4	17.1	17.0	16.1	-	-	-	-	-	-	-	-	-	-	-	-	-	-	-	-	-	-	-
A7	17.9	15.1	9.1	17.1	-	-	-	-	-	-	-	-	-	-	-	-	-	-	-	-	-	-
A8	17.5	17.1	16.3	17.3	16.0	-	-	-	-	-	-	-	-	-	-	-	-	-	-	-	-	-
A9	16.6	15.5	13.6	16.2	15.4	15.1	-	-	-	-	-	-	-	-	-	-	-	-	-	-	-	-
A11	17.1	16.1	15.5	18.3	16.2	16.9	14.9	-	-	-	-	-	-	-	-	-	-	-	-	-	-	-
A12	17.4	12.2	13.1	17.0	13,2	16.9	16.7	14.9	-	-	-	-	-	-	-	-	-	-	-	-	-	-
A13	16.2	11.6	13.3	15.2	15.0	17.0	16.2	14.8	13.5	-	-	-	-	-	-	-	-	-	-	-	-	-
A16	18.8	15.5	17.4	16.6	17.9	19.2	19.3	18.1	16.7	16.1	-	-	-	-	-	-	-	-	-	-	-	-
A17	15.6	12.6	11.0	14.7	11.9	16.4	12.9	16.2	12.0	14.2	15.6	-	-	-	-	-	-	-	-	-	-	-
A18	19.0	13.8	15.3	17.4	16.0	18.8	17.2	17.8	15.3	13.0	16.5	14.4	-	-	-	-	-	-	-	-	-	-
A19	16.9	13.9	12.1	14.4	14.2	14.8	5.1	14.1	15.4	14.5	17.4	11.6	15.6	-	-	-	-	-	-	-	-	-
A20	18.9	14.9	15.4	17.6	14.4	18.6	17.3	16.1	15.1	15.9	16.2	16.1	16.7	15.4	-	-	-	-	-	-	-	-
A21	16.3	15.1	14.3	18.2	14.9	18.6	16.5	15.0	14.5	14.0	16.8	15.4	15.4	16.1	16.7	-	-	-	-	-	-	-
A22	22.4	19.7	18.9	19.9	20.9	21.5	20.1	21.2	20.1	20.4	21.4	19.0	18.8	19.8	20.5	20.2	-	-	-	-	-	-
5560	16.0	15.1	12.5	13.9	14.2	18.1	14.3	16.2	15.4	15.1	16.1	12.8	17.2	12.0	13.9	15.1	16.6	-	-	-	-	-
HE2	16,7	15.6	13.3	15.9	13.7	18.5	14.3	17.4	15.1	13.2	17.1	11.9	13.5	13.5	15.9	13.1	19.2	10.3	-	-	-	-
BY1	16.2	14.9	14.1	15.5	14.4	17.8	14.0	17.4	15.0	13.6	16.6	12.2	14.5	13.2	14.9	14.3	18.4	9.9	2.2	-	-	-
NW1	17.9	14.7	14.9	15.9	15.4	18.8	16.8	14.1	15.9	15.1	14.7	15.9	14.8	16.6	15.6	12.8	19.3	12.5	14.7	14.7	-	-
B7	17.0	12.5	11.5	15.7	13.7	16.7	14.9	14.7	14.2	11.3	16.3	11.0	11.6	12.0	15.9	14.8	18.8	12.5	12.3	13.5	14.9	-
**A5**	**18.1**	**15.0**	**14.7**	**15.6**	**15.2**	**16.0**	**15.9**	**17.1**	**15.4**	**15.3**	**16.8**	**14.4**	**17.8**	**14.5**	**16.4**	**16.4**	**18.5**	**8.4**	**11.3**	**10.6**	**14.4**	**13.2**

## Data Availability

All data generated and analyzed in this study are included in this published article.
